# Distinct Evolutionary Constraints Shape TNL and CNL Immune Receptors in Wild and Cultivated Tomato

**DOI:** 10.3390/biology15161350

**Published:** 2026-08-10

**Authors:** Shibo Meng, Jiajun Zhu, Enmei Hu, Jia Liu, Yuan Cheng, Meiying Ruan, Chenxu Liu, Qingjing Ye, Rongqing Wang, Zhuping Yao, Zhimiao Li, Guozhi Zhou, Hongjian Wan, Yougen Chen

**Affiliations:** 1 College of Horticulture, Anhui Agricultural University, Hefei 230036, China; 24720187@stu.ahau.edu.cn (S.M.); 24721214@stu.ahau.edu.cn (J.Z.); 2 State Key Laboratory for Quality and Safety of Agro-Products, Institute of Vegetables, Zhejiang Academy of Agricultural Sciences, Hangzhou 310021, China; liu-l-jia@163.com (J.L.); chengyuan@zaas.ac.cn (Y.C.); ruanmy@zaas.ac.cn (M.R.); liuchenxu@zaas.ac.cn (C.L.); yeqj@zaas.ac.cn (Q.Y.); wangrq@zaas.ac.cn (R.W.); yaozp@zaas.ac.cn (Z.Y.); zhimiaoli@zaas.ac.cn (Z.L.); zhougz@zaas.ac.cn (G.Z.); 3 Department of Agronomy and Horticulture, Jiangsu Vocational College of Agriculture and Forestry, Jurong 212400, China; xfx202202@jsafc.edu.cn

**Keywords:** NLR gene family, NB-ARC domain, Ka/Ks selection pressure, disease resistance breeding, pattern-triggered immunity

## Abstract

Nucleotide-binding site leucine-rich repeat (NLR, historically termed NBS-LRR) proteins are among the most rapidly evolving components of plant innate immunity, yet comparative analyses of NLR subfamily diversification between wild and cultivated accessions of tomato (*Solanum lycopersicum*) have remained limited. Here, we compared the NLR gene family across three representative tomato accessions—the wild species *S. chilense* and the cultivated tomatoes *S. lycopersicum* Heinz1706 (large-fruited) and *S. lycopersicum* LA1464 (cherry tomato)—by integrating genome-wide identification, phylogenetic reconstruction, motif analysis, orthologous clustering, selection-pressure assessment, and expression profiling. We identified 220, 223, and 245 candidate NLR genes in *S. chilense*, Heinz1706, and LA1464, respectively, and classified them into TIR-type (TNL) and coiled-coil-type (CNL) subfamilies at a consistent ratio of approximately 1:5 across all three accessions. Motif analysis revealed pronounced structural divergence between TNL and CNL proteins, most notably in the region corresponding to the second conserved motif of the NB-ARC domain. Orthologous clustering identified 67 gene clusters shared among the three accessions; within these, TNL genes showed greater sequence divergence (higher Ka and Ks values) than CNL genes but significantly lower Ka/Ks ratios (ω), indicating stronger purifying selection despite their greater raw divergence. Expression profiling further showed that CNL genes were more broadly expressed across tissues and were induced by multiple pathogen-associated molecular patterns, whereas TNL gene expression was more spatially restricted and preferentially induced by effector-related treatments. These results indicate that the TNL and CNL subfamilies of the tomato NLR repertoire have followed distinct evolutionary trajectories and regulatory strategies. As these conclusions are based on comparative genomic and transcriptomic evidence from three accessions rather than on functional validation, they should be regarded as hypotheses; nonetheless, they offer candidate genomic resources and a comparative framework that may inform future disease-resistance breeding in tomato.

## Simple Summary

Plants defend themselves against pathogens using large families of immune receptor genes. In tomato, these genes fall into two main groups, called TNL and CNL, which are thought to detect pathogens in different ways. It has not been clear how these two groups differ in their evolution and behavior, particularly when wild tomato relatives are compared with the cultivated varieties grown by farmers. In this study, we compared the immune receptor genes of one wild tomato species and two cultivated tomato varieties using publicly available genome and gene-expression data. We found that CNL-type genes were far more numerous than TNL-type genes in all three tomatoes and that the two groups differed in protein structure, in how quickly their gene sequences changed over evolutionary time, and in which tomato tissues and conditions activated them. TNL genes changed more slowly and appeared to be tightly conserved for a core immune function, whereas CNL genes evolved faster and were switched on more broadly, consistent with defending against a wider range of pathogens. These findings, which are based on computational comparisons of public data rather than new laboratory experiments, provide a useful starting point for researchers and breeders seeking to identify tomato genes that could help improve disease resistance in future tomato varieties.

## 1. Introduction

Plant nucleotide-binding site leucine-rich repeat (NBS-LRR), also known as NLR, proteins constitute one of the largest classes of intracellular immune receptors in plants. These proteins play a central role in plant innate immunity by recognizing pathogen-derived effector proteins and activating downstream defense responses [1]. Typical NBS-LRR proteins share a conserved modular architecture composed of a variable N-terminal domain, a central nucleotide-binding site (NBS, or NB-ARC) domain, and a C-terminal leucine-rich repeat (LRR) domain [2,3]. The NB-ARC domain functions as a molecular switch through ATP/ADP binding and hydrolysis, cycling between an ADP-bound inactive state and an ATP-bound active state, whereas the LRR domain contributes to effector recognition via direct or indirect interactions with pathogen proteins [4].

Based on variation in their N-terminal domains, NBS-LRR proteins are classified into two major subclasses: TIR-NBS-LRR (TNL) proteins, containing a Toll/interleukin-1 receptor (TIR) domain, and non-TIR-NBS-LRR (nTNL) proteins, which lack this domain. Most nTNL proteins possess an N-terminal coiled-coil (CC) domain and are therefore referred to as CC-NBS-LRR (CNL) proteins, while a smaller subset lacks a recognizable N-terminal domain and is classified as NOD-like receptor (NL, NBS-LRR without a recognizable N-terminal domain) [5,6]. NBS-LRR genes are typically organized in clusters within plant genomes, a feature that promotes gene duplication, recombination, and unequal crossing over, thereby accelerating gene diversification and the evolution of novel resistance specificities [7]. This clustered genomic arrangement is thought to be biologically important because it increases the local mutational and recombinational opportunities available to the gene family, allowing individual paralogs to diversify rapidly; this is widely considered a key mechanism by which plant genomes keep pace with the rapid evolution of pathogen effectors and maintain durable disease resistance over evolutionary time.

Comparative genomic analyses have revealed pronounced lineage-specific differences in the abundance and composition of NBS-LRR subclasses. In many dicotyledonous plants, including *Arabidopsis thaliana*, cassava, potato, and tobacco, nTNL genes are more abundant than TNL genes [8,9,10]. In contrast, *Arabidopsis lyrata* and soybean harbor expanded TNL repertoires, with TNL genes outnumbering CNL genes by several-fold [11,12]. In monocotyledonous plants such as rice, TNL genes are nearly absent, and the NBS-LRR family is dominated by nTNL members, predominantly CNLs [13,14]. Together, these comparative genomic analyses across Arabidopsis, rice, soybean, cassava, potato, and other crops reveal substantial lineage-specific expansion and contraction of NLR repertoires, reflecting diverse evolutionary strategies shaped by pathogen pressure, highlighting distinct evolutionary trajectories of plant immune receptors.

NBS-LRR proteins confer resistance to a wide range of pathogens, including bacteria, fungi, viruses, oomycetes, nematodes, and insects, through either direct effector recognition or indirect surveillance mechanisms described by the “guard hypothesis” [15,16].

TNL proteins are characterized by an N-terminal TIR domain that is evolutionarily conserved across plants, animals, and microorganisms and plays a pivotal role in immune signal transduction [17]; some TNL proteins additionally contain a C-terminal jelly-roll/Ig-like domain (C-JID) that cooperates with the LRR domain in effector recognition [18,19]. In contrast, nTNL proteins, most of which carry a CC domain (CNL), signal through CC-mediated oligomerization into resistosome complexes upon effector perception [20]—exemplified by the Arabidopsis CNL protein ZAR1 [21], whose resistosome forms a plasma membrane-inserted calcium channel that triggers Ca^2+^ influx, reactive oxygen species production, and programmed cell death [22,23,24].

Tomato (*Solanum lycopersicum*), an economically important Solanaceae crop, has served as a key model for investigating NBS-LRR-mediated disease resistance. In tomato, several NLR genes, including Mi-1.2, Sw-5, and I2, have been functionally characterized and confer resistance against root-knot nematodes, tospoviruses, and the vascular wilt fungus *Fusarium oxysporum* f. sp. *lycopersici*, respectively, illustrating the functional diversity of NLR-mediated immunity in this crop [25,26,27]. Genome-wide analyses have identified between approximately 250 and 320 NBS-LRR genes in tomato, depending on genome versions and identification criteria [7,28]. Comparative studies involving cultivated tomato and its wild relatives consistently demonstrate a strong subclass bias toward nTNL genes, particularly CNLs, whereas TNL genes account for only a small proportion of the total NBS-LRR repertoire [29]. However, these studies have primarily reported subclass abundance without directly contrasting the evolutionary constraints, structural features, and expression behavior of the TNL and CNL subfamilies within a single comparative framework, and without explicitly considering how domestication may have shaped this composition. The structural organization, evolutionary dynamics, and functional diversification of tomato NBS-LRR genes therefore remain incompletely understood, underscoring the need for an integrative analysis that jointly evaluates wild and cultivated genomes.

Although existing studies have analyzed the NBS-LRR gene family in tomatoes, most have focused on single cultivars and have not directly compared wild and cultivated genomes within the same analytical framework [30,31], particularly with respect to the evolutionary constraints—evaluated here primarily through pairwise Ka/Ks (nonsynonymous/synonymous substitution) ratios—that act differentially on the TNL and CNL subfamilies. To address this gap, we selected three tomato accessions representing different points along the domestication continuum: the wild species *S. chilense*, which has not been subject to human selection; *S. lycopersicum* Heinz1706, a highly domesticated, large-fruited processing cultivar; and *S. lycopersicum* LA1464, a cherry-type cultivar representing an earlier stage of tomato domestication. We hypothesized that, owing to differences in their signaling mechanisms and breadth of pathogen recognition, the TNL and CNL subfamilies have been subject to distinct evolutionary constraints and regulatory strategies and that these differences are further modulated by domestication. Using genome-wide identification, phylogenetic reconstruction, motif analysis, orthologous clustering, selection-pressure assessment, and expression profiling, this comparative analysis—while based on computational prediction across a limited sample of three accessions rather than on direct functional characterization—provides new insight into the evolutionary and regulatory divergence between the two classes of tomato immune receptors and into how domestication may have shaped the composition of the tomato immune receptor repertoire.

## 2. Materials and Methods

### 2.1. Data Sources and Processing

Three representative tomato accessions were selected for this study, including the wild tomato *S. chilense*, the cultivated large-fruited tomato *S. lycopersicum* Heinz1706, and the cultivated cherry tomato *S. lycopersicum* LA1464. Genome assemblies and corresponding annotation files for these accessions were obtained from publicly available databases, including the CAAS Tomato Pan-genome (http://caastomato.biocloud.net/home [accessed on 18 June 2025]) and TomatoPangenome (https://www.varnatech.cn/tomatoPan) resources (accessed on 18 June 2025); because these resources are updated periodically, the specific genome version used for each accession is unambiguously fixed by the RefSeq accession numbers reported below. All genome datasets were subjected to quality assessment to ensure their completeness and suitability for genome-wide gene family analyses, assessed with BUSCO (v5.4.3, solanales_odb10 lineage dataset); only assemblies with a BUSCO complete-ortholog score above 90% were retained, and annotation quality was further checked by confirming consistency between the predicted gene models and the corresponding RefSeq annotation release. These three accessions were selected because they represent, respectively, an undomesticated wild relative, a highly domesticated large-fruited cultivar, and a less intensively selected cherry-type cultivar, each with a high-quality, well-annotated genome assembly, thereby enabling comparison across the tomato domestication gradient; we note that residual differences in assembly contiguity or annotation completeness among the three genomes, despite passing the same quality thresholds, could contribute marginally to differences in the number of NBS-LRR genes identified, a possibility considered further in the Discussion. Prior to domain searches, protein sequences were filtered to retain only the longest predicted transcript per gene locus. The Ref-Seq accession numbers, retrieved from the NCBI Ref-Seq database, for the genome assembly and annotation data of the three tomato materials are as follows: *S. chilense* (GCF_000499545.2), *S. lycopersicum* Heinz1706 (GCF_000188115.4), and *S. lycopersicum* LA1464 (GCF_000390615.1).

### 2.2. Identification of NBS-LRR Genes

Candidate NBS-LRR genes were initially identified by searching the predicted proteomes of the three tomato accessions using the hmmsearch program implemented in the HMMER suite (v3.3.2) [32]. Searches were performed using the NB-ARC domain hidden Markov model (HMM) profile PF00931 retrieved from the Pfam database (https://www.ebi.ac.uk/interpro/entry/pfam/PF00931/ [accessed on 3 July 2025]). An E-value cutoff of 0.001, a threshold widely used in HMMER-based domain searches to balance sensitivity and specificity for high-confidence NB-ARC domain detection, was applied to ensure high-confidence detection. Duplicate gene models and putative pseudogenes (sequences containing premature stop codons or frameshifts) were removed prior to domain validation. To confirm domain integrity, all candidate proteins were further validated using the SMART9.0 online platform (http://smart.embl.de/ [accessed on 20 July 2025]), and only genes containing a complete NB-ARC domain were retained for subsequent analyses. We note that this domain-centered identification strategy prioritizes specificity but may under-represent NBS-LRR genes with highly divergent or partial NB-ARC domains, a limitation considered further in the Discussion. Genes were subsequently assigned to the TNL, CNL, or NL subfamily according to the presence or absence of an N-terminal TIR or CC domain, identified by SMART9.0 annotation together with phylogenetic clustering against reference sequences (Section 2.3); proteins lacking a recognizable N-terminal domain were classified as NL.

### 2.3. Phylogenetic Analysis

To infer the phylogenetic relationships among tomato NBS-LRR genes, experimentally validated NBS-LRR reference sequences were retrieved from the PRGdb4.0 database (https://prgdb.org/prgdb4/ [accessed on 22 July 2025]), including the TNL-type gene PRGDB131, the CNL-type gene PRGDB140, and the NL-type gene PRGDB141, chosen because they are experimentally validated, well-characterized representatives of each NBS-LRR subclass that are commonly used as classification anchors in comparative NBS-LRR studies; we acknowledge that a single reference sequence per subclass may not fully capture the internal diversity of each subfamily. Multiple sequence alignment of the extracted NB-ARC domain sequences (rather than full-length proteins) was performed using ClustalX2.1 [33], and alignments were visually inspected to confirm correct alignment of the conserved P-loop, Kinase-2, and GLPL motifs; alignment termini were not further trimmed. Phylogenetic trees were then constructed using the Neighbor-Joining (NJ) method implemented in MEGA11.0.13 software. Genetic distances were estimated using the p-distance model, assuming uniform evolutionary rates among sites, an approach chosen for its computational efficiency in the large-scale, exploratory subclass assignment that was the goal of this analysis rather than fine-scale phylogenetic resolution; we recognize that maximum-likelihood or Bayesian methods with explicit substitution models would offer higher statistical accuracy for detailed evolutionary inference and recommend their use in future, more fine-grained phylogenetic analyses of this gene family. The robustness of tree topology was assessed by 1000 bootstrap replicates, and missing data were treated using the pairwise deletion option [34].

### 2.4. Conserved Motif Analysis

Conserved motif analysis of NBS-LRR proteins from the three tomato accessions was conducted using the MEME5.5.3 online suite (https://meme-suite.org/meme/ [accessed on 25 July 2025]), applied to the extracted NB-ARC domain region rather than full-length protein sequences, consistent with the alignment strategy described above. The maximum number of motifs was set to 10, based on preliminary runs in which additional motifs beyond this number were increasingly short, of low statistical support, or redundant with motifs already identified, while all other parameters were maintained at default settings (zero-or-one-occurrence-per-sequence [zoops] site distribution, minimum and maximum motif widths of 6 and 50 residues, respectively, and a motif E-value significance threshold of 0.05). Identified motifs and their positional distributions were visualized using TBtools1.12 software [35], with particular emphasis on comparing motif composition between the TNL and CNL subfamilies. Identified motifs were additionally compared against previously described conserved NB-ARC motifs (e.g., P-loop, Kinase-2, GLPL, MHD) to assign putative functional identities where possible.

### 2.5. Orthologous Clustering Analysis

Orthologous clustering of NBS-LRR protein sequences from the three tomato accessions was performed using the OrthoVenn3 online platform (https://orthovenn3.bioinfotoolkits.net/ [accessed on 30 July 2025]). The analysis was conducted with an E-value threshold of 1 × 10^−5^ and an inflation parameter of 1.5, values within the default/recommended range of OrthoVenn3 for identifying orthologous clusters among moderately divergent genomes and commonly adopted in comparative plant gene-family studies. Clusters containing sequences from all three accessions were treated as conserved (core) orthogroups, whereas clusters restricted to one or two accessions were considered accession-specific or partially shared, respectively. Only the complete NBS-LRR protein sequences retained after the filtering described in Section 2.2 were included; partial sequences were excluded. The resulting orthologous clusters were visualized as Venn diagrams and heatmaps to illustrate conserved and accession-specific NBS-LRR genes, and cluster size was used as a descriptive proxy for gene-family expansion or contraction among accessions; we acknowledge that no additional formal statistical test of cluster-size differences was performed, which is considered a limitation of this analysis.

### 2.6. Selection Pressure Analysis

Orthologous gene pairs belonging to the TNL and CNL subfamilies were identified from shared gene clusters, defined as reciprocal best matches within the shared orthologous clusters identified in Section 2.5; the corresponding coding sequences were then aligned in a codon-aware manner guided by the protein alignment, and pairs with Ks values indicative of substitution saturation (Ks > 2) were excluded from downstream analysis. Corresponding coding sequences (CDSs) and protein sequences were extracted for evolutionary analysis. The nonsynonymous substitution rate (Ka), synonymous substitution rate (Ks), and their ratio (ω = Ka/Ks) were calculated using the “Simple Ka/Ks Calculator (NG)” module integrated in TBtools1.12 [35], which implements the Nei–Gojobori method; this method was selected for its computational simplicity and wide use in large-scale, exploratory comparisons of gene-family evolution. We recognize that codon-based maximum-likelihood approaches (e.g., PAML (https://github.com/abacus-gene/paml/releases), KaKs_Calculator 2.0, HyPhy (http://hyphy.org/)), which can accommodate heterogeneous substitution rates among sites and codon positions, provide more statistically robust estimates and represent a valuable direction for future, more detailed evolutionary analyses of this gene family. Distribution patterns of Ka, Ks, and ω values were visualized using Origin2025 (OriginLab, Northampton, MA, USA) in the form of violin plots combined with boxplots. Differences in the distributions of Ka, Ks, and ω between TNL and CNL genes were evaluated using the Mann–Whitney U test, given the non-normal distribution of these values, with statistical significance set at *p* < 0.05, in addition to the PGLS-based correction for phylogenetic non-independence described in Section 2.8.

### 2.7. Expression Analysis

RNA-seq expression data were obtained from the Tomato Functional Genomics Database (TFGD; http://ted.bti.cornell.edu [accessed on 16 August 2025]), specifically from the Digital Expression Database. Expression levels of TNL and CNL genes in *S. lycopersicum* Heinz1706 were extracted and quantified as reads per kilobase per million mapped reads (RPKM) across multiple tissues, developmental stages, and pathogen treatment conditions. Genes with an average expression level greater than 1 RPKM were retained for further analysis, a conventional cutoff used to exclude genes whose expression cannot be reliably distinguished from background noise, although we acknowledge that it may also exclude some biologically relevant, lowly expressed resistance genes, and expression profiles of their homologous genes were examined in the wild accession *S. pimpinellifolium* LA1589. We note that LA1589 is an accession of *S. pimpinellifolium*, a wild tomato line closely related to, but taxonomically distinct from, *S. chilense* within the tomato wild-relative complex; LA1589 was used here rather than *S. chilense* itself because it is the wild accession with the most comprehensive expression data currently available in TFGD. We acknowledge that this substitution is an important limitation: expression patterns in *S. pimpinellifolium* LA1589 may not fully reflect those of *S. chilense*, and this limitation is discussed further below. Differences in expression between corresponding tissues or treatments were not subjected to formal statistical testing beyond RPKM thresholding and Z-score visualization, and potential batch effects between the Heinz1706 and LA1589 datasets were not explicitly corrected; these are noted as limitations of the present expression analysis. The expression matrix was normalized by rows (Z-score transformation) using TBtools1.12, and heatmaps were generated to visualize expression variation under different biological conditions; because this transformation emphasizes the relative pattern of expression across conditions rather than absolute expression differences, the underlying RPKM values (Table S5) should be consulted when interpreting the magnitude of expression differences between genes or accessions.

### 2.8. Statistical Analysis and Visualization

All statistical analyses and data visualizations were performed using the aforementioned bioinformatics tools, including TBtools1.12 and Origin2025 [35]. These analyses were conducted following standardized workflows to ensure reproducibility and clarity of data presentation. Differences in Ka, Ks, and ω distributions between TNL and CNL genes were assessed using the Mann–Whitney U test after confirming departure from normality with the Shapiro–Wilk test; where multiple pairwise comparisons were performed, *p*-values were adjusted using the Benjamini–Hochberg procedure to control the false discovery rate. Because NBS-LRR genes often exist in the form of gene clusters and exhibit phylogenetic non-independence, we corrected the results of selection pressure analysis using a phylogenetic generalized least squares (PGLS) model. We used a phylogenetic tree constructed with MEGA11 as input and performed the analysis using the caper package in R, specifying a Brownian-motion model of trait evolution, with Ka/Ks (ω) as the response variable, the TNL/CNL subfamily identity as the predictor, and the NJ tree from Section 2.3 as the input topology; parameter estimates, 95% confidence intervals, and significance thresholds (*p* < 0.05) are reported in the Results. This PGLS-based correction was applied specifically to eliminate the impact of phylogenetic non-independence on the statistical results.

## 3. Results

### 3.1. Multi-Variety Genome Comparison and NBS-LRR Gene Identification

A reference genome represents the genetic composition of a single individual and therefore cannot fully capture the genetic diversity present within a species. Due to adaptive evolution and environmental selection, substantial genomic variation, including copy number variation (CNV) and presence–absence variation (PAV), exists among different subspecies and accessions. To better represent population-level diversity, three tomato accessions with high-quality genome assemblies and comprehensive gene annotations were selected for comparative analysis: the wild tomato species *S. chilense*, the cultivated large-fruited tomato *S. lycopersicum* Heinz1706, and the cultivated cherry tomato *S. lycopersicum* LA1464. Using the NB-ARC domain HMM profile (PF00931) from the Pfam database, hmmsearch was applied to identify NBS-LRR genes in the three tomato genomes. With a stringent E-value cutoff of <0.001, a total of 220, 223, and 245 putative NBS-LRR genes were identified in *S. chilense*, Heinz1706, and LA1464, respectively (Table S1). Consistent with previous reports, these genes were distributed across all 12 chromosomes as well as unanchored scaffolds in the tomato genome.

### 3.2. Phylogenetic Classification Based on the NB-ARC Domain

Conserved domains are protein segments that remain highly preserved throughout evolution and are commonly used for gene family classification. In NBS-LRR proteins, the NB-ARC domain represents the central conserved region and was therefore used as the basis for phylogenetic analysis. During initial identification, incomplete genome annotation and gene fragmentation were detected in all three tomato genomes, resulting in the presence of partial NBS-LRR sequences lacking complete conserved domains. Only genes containing a complete NB-ARC domain were retained. After filtering, 176 complete NBS-LRR genes were identified in *S. chilense*, 154 in *S. lycopersicum* Heinz1706, and 173 in *S. lycopersicum* LA1464 (Table S2). The proportion of genes retained after filtering differed among accessions, reflecting discrepancies between initial candidate numbers and validated sequences containing complete NB-ARC domains. Multiple sequence alignment of NB-ARC domains showed that conserved motifs, including the P-loop, Kinase-2, and GLPL regions, were present across all retained sequences. Sequence length and composition of the NB-ARC domains were comparable among the three accessions, providing a uniform dataset for phylogenetic reconstruction.

### 3.3. Subfamily Distribution of TNL, CNL, and NL Genes

**Figure 1:**
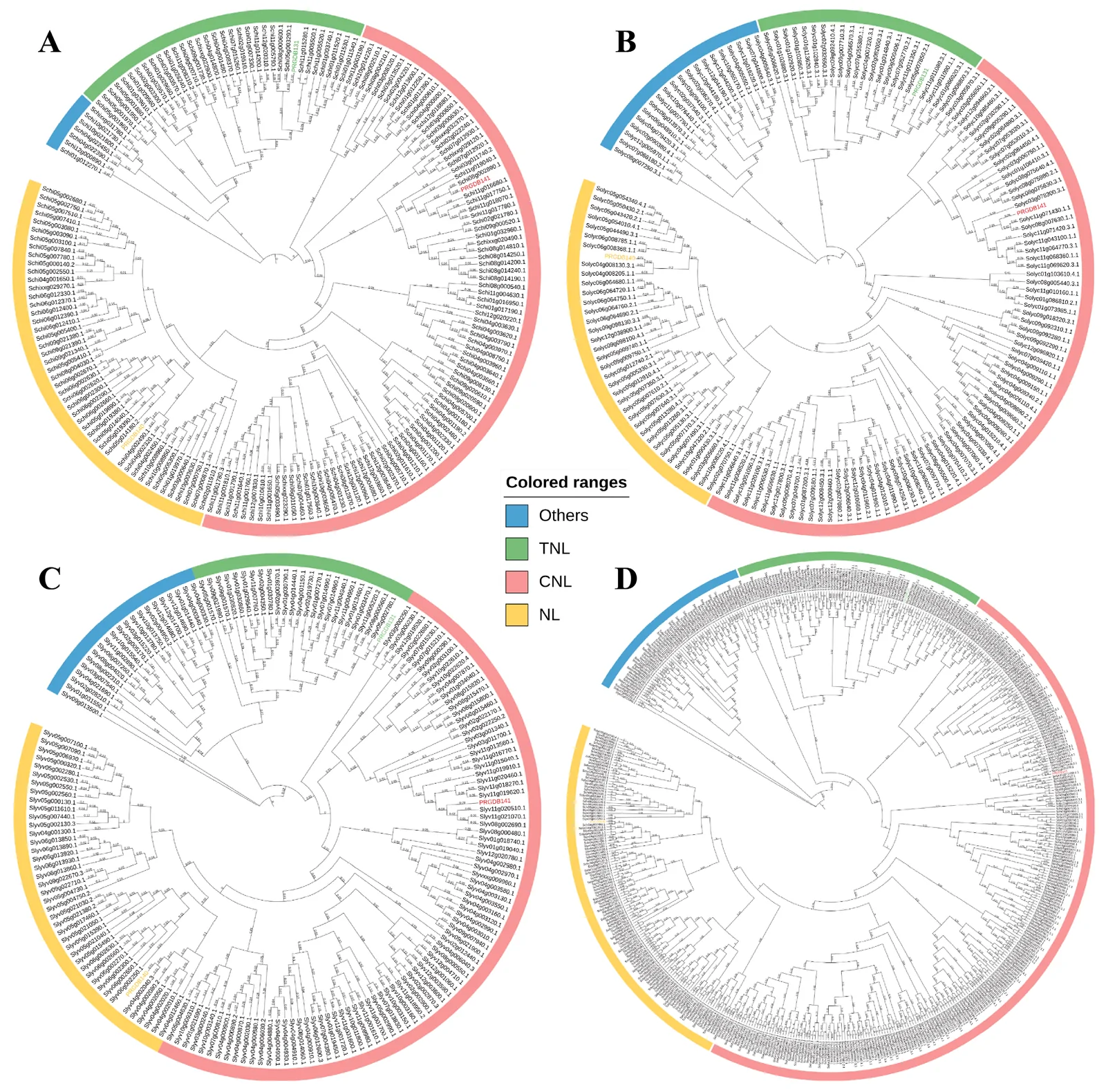
Phylogenetic tree of NBS-LRR genes from three tomato varieties, individually and collectively, generated by clustering using conserved domains. TNL reference gene PRGDB131 is green, CNL reference gene PRGDB140 is red, and NL reference gene PRGDB141 is yellow. The green outer ring represents TNL genes, the red outer ring represents CNL genes, the yellow outer ring represents NL genes, and the blue outer ring represents genes containing other atypical domains. (**A**) *S. chilense*, (**B**) *S. lycopersicum* Heinz1706, (**C**) *S. lycopersicum* LA1464, (**D**) Three tomato species combined.

To classify NBS-LRR genes into subfamilies, experimentally validated tomato NBS-LRR reference sequences from PRGdb 4.0 were included in the analysis (TNL: PRGDB131; CNL: PRGDB140; NL: PRGDB141). Conserved NB-ARC domains from reference and candidate genes were aligned using ClustalX2.1, and phylogenetic trees were constructed using the neighbor-joining method with 1000 bootstrap replicates. Phylogenetic trees generated for individual accessions and the combined dataset showed clear clustering of reference and candidate genes into TNL, CNL, and NL branches (Figure 1). Subfamily assignments inferred from phylogenetic clustering were further verified using SMART9.0 domain annotation. The final numbers of TNL, CNL, and NL genes for each accession are summarized in Table 1. Across the three tomato accessions, nTNL genes were more abundant than TNL genes. The ratio of nTNL to TNL genes was approximately 5:1 in all accessions. CNL genes constituted the largest proportion of the NBS-LRR gene family, while NL genes accounted for a smaller fraction. The relative proportions of TNL, CNL, and NL genes were similar among *S. chilense*, Heinz1706, and LA1464, despite differences in total gene number.

### 3.4. Conserved Motif Composition of TNL and nTNL Proteins

**Figure 2:**
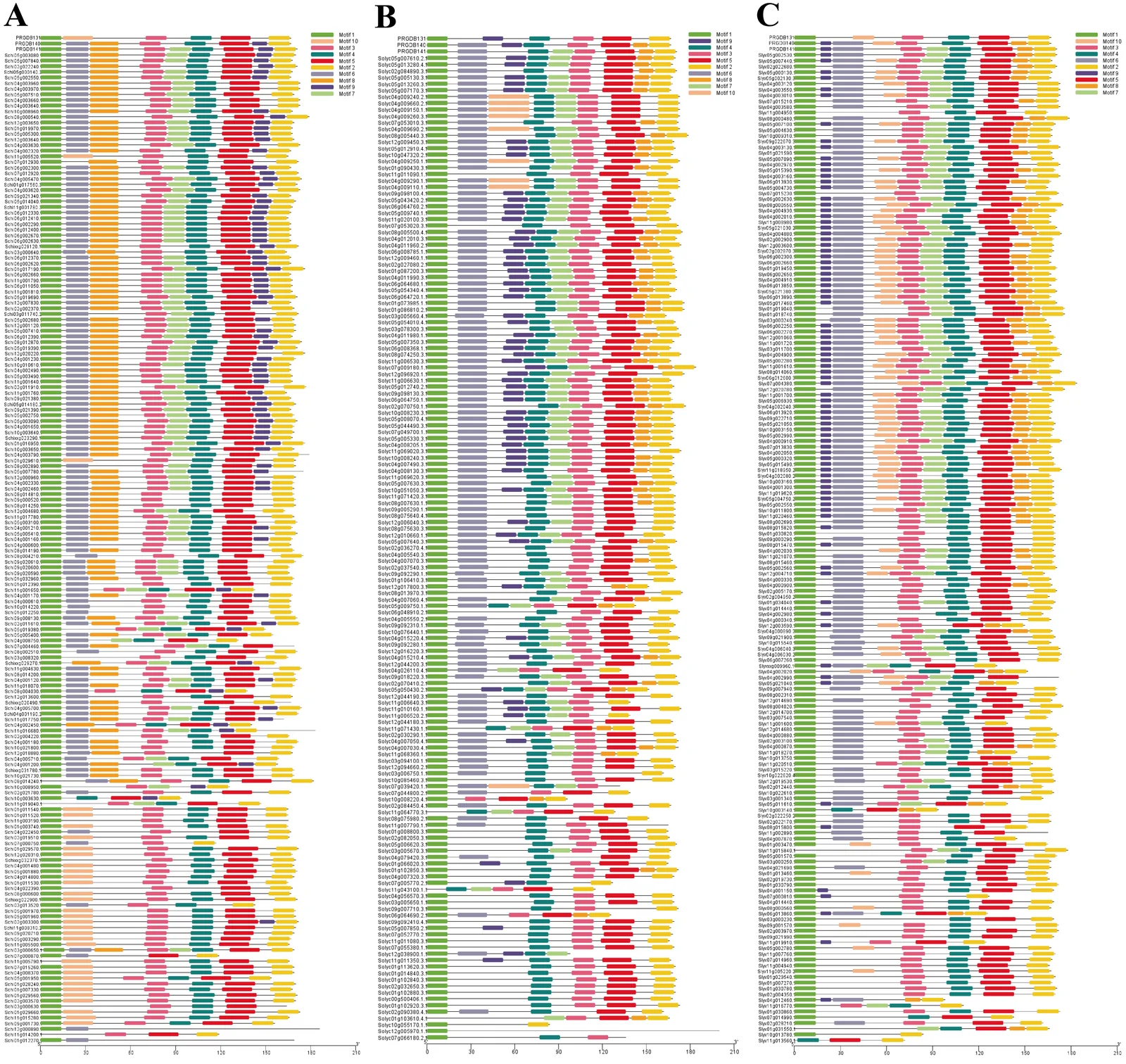
Visualization of conserved domain motifs of NBS-LRR genes in three tomato species, with a maximum of 10 motifs set for identification. PRGDB131 (TNL), PRGDB140 (CNL), and PRGDB141 (NL) serve as representative reference genes. (**A**) *S. chilense*, (**B**) *S. lycopersicum* Heinz1706, (**C**) *S. lycopersicum* LA1464. Note: The figure shows the motif structures of 10 randomly selected proteins from each material. Because the total number of protein motifs differs among materials from different varieties, different representative motifs may have been selected in different varieties for the same reference gene. The protein motif structure difference diagram is only used to compare structural differentiation among subfamilies within a variety and structural variation within the same subfamily. These results are helpful for understanding the functional differentiation of different immune receptors.

To examine conserved motif composition, MEME5.5.3 was used to identify motifs within the NB-ARC domains of NBS-LRR proteins, with the number of motifs set to 10. Representative PRGdb reference genes were included to assist motif annotation. Motif positions and distributions were visualized using TBtools1.12 (Figure 2). CNL proteins displayed a relatively uniform motif arrangement across the three accessions, with most identified motifs consistently present and aligned in a similar order along the NB-ARC domain. In contrast, TNL proteins showed variation in motif composition. Specifically, TNL proteins in *S. chilense* lacked motifs 6, 8, and 9 but contained motif 10. In Heinz1706, TNL proteins lacked motif 6 but retained motif 10. In LA1464, TNL proteins lacked motifs 5, 8, and 9. The motifs absent in TNL proteins were consistently located downstream of motif 1. Motif 10 was detected in TNL proteins of *S. chilense* and Heinz1706 but was not observed in LA1464. The detailed information of 10 conserved motifs identified by MEME in three *S. lycopersicum* accessions is shown in Table S3.

### 3.5. Orthologous Gene Similarity and Selection Pressure Analysis

**Figure 3:**
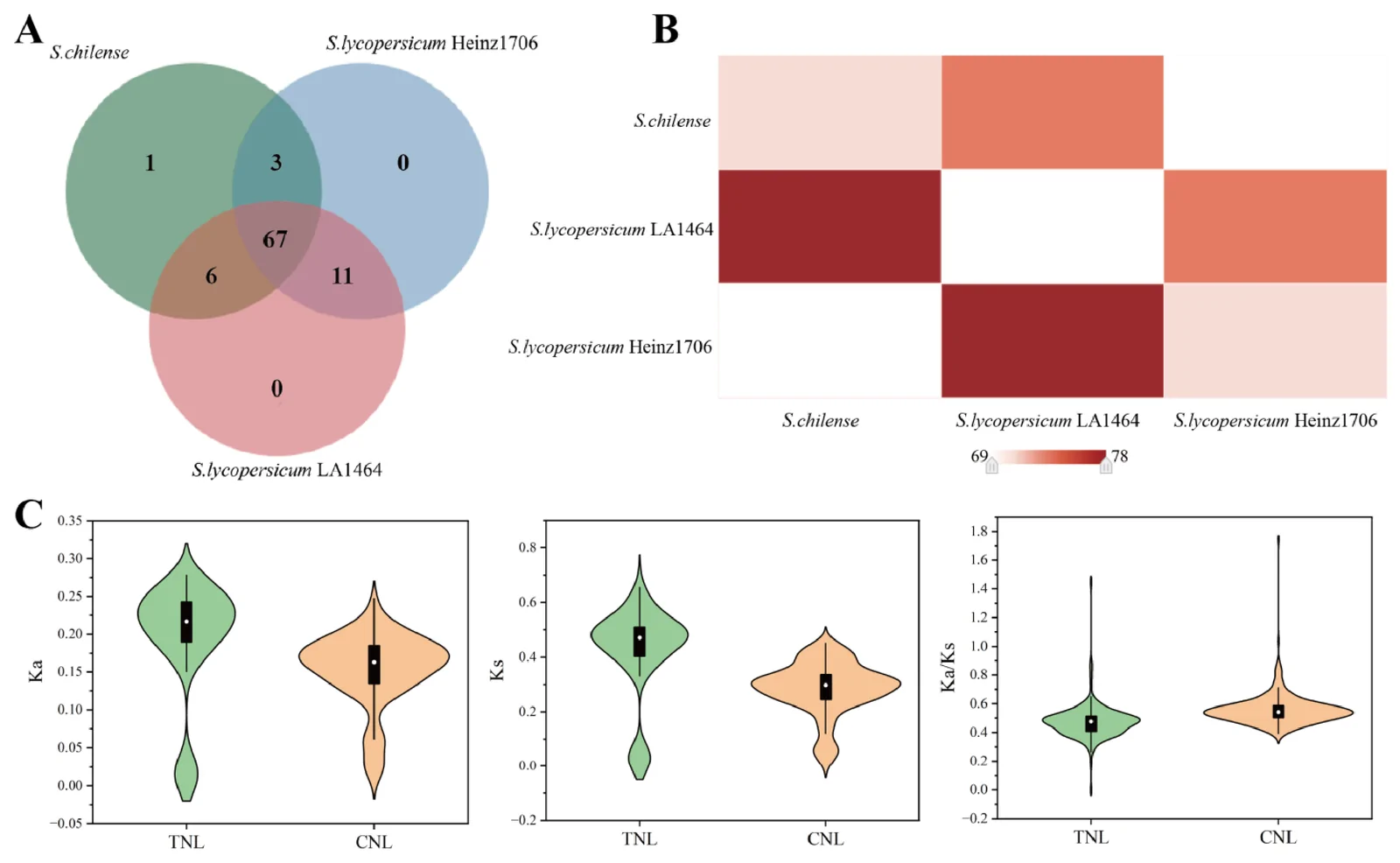
Visualization of similarity and evolutionary pressure of genes from three tomato species. (**A**) Venn diagram of NBS-LRR orthologous gene clusters from three tomato species. (**B**) Heatmap showing the number of NBS-LRR orthologous gene clusters pairwise among the three tomato species, with colors from light to dark representing quantities from few to many. (**C**) Violin plots with box plots of Ka, Ks, and Ka/Ks for orthologous TNL and CNL gene clusters from three tomato species. The box plots show the median (Q2, white dot) and interquartile range (Q1–Q3, black box), with whiskers extending to 1.5 × IQR.

Orthologous clustering of NBS-LRR proteins from the three tomato accessions was performed using OrthoVenn3. The resulting Venn diagram and heatmap (Figure 3A,B) showed that Heinz1706 and LA1464 shared a larger number of orthologous gene clusters compared with *S. chilense*. Among the cultivated accessions, sequence similarity was higher between Heinz1706 and LA1464 than between either cultivated accession and *S. chilense*. *S. chilense* shared more orthologous gene clusters with LA1464 than with Heinz1706. After excluding accession-specific genes, 67 orthologous NBS-LRR gene clusters were shared among all three accessions. These clusters were classified into TNL and CNL subfamilies based on SMART9.0 domain annotation. Coding sequences from each orthologous cluster were extracted, and Ka, Ks, and Ka/Ks (ω) values were calculated using TBtools1.12 (Table S4). The distributions of Ka, Ks, and ω values for TNL and CNL genes are shown in Figure 3C. Median Ka and Ks values were higher for TNL genes than for CNL genes. In contrast, median ω values were lower for TNL genes than for CNL genes. Even after correction using the PGLS model, our findings continued to demonstrate that the Ka/Ks values of TNL genes were significantly lower than those of CNL genes, suggesting the robustness of our results against phylogenetic non-independence and affirming the reliability of our conclusions. Individual gene pairs showed variation in all three parameters within each subfamily.

### 3.6. Differential Expression of TNL and CNL Genes Under Developmental and Immune Conditions

**Figure 4:**
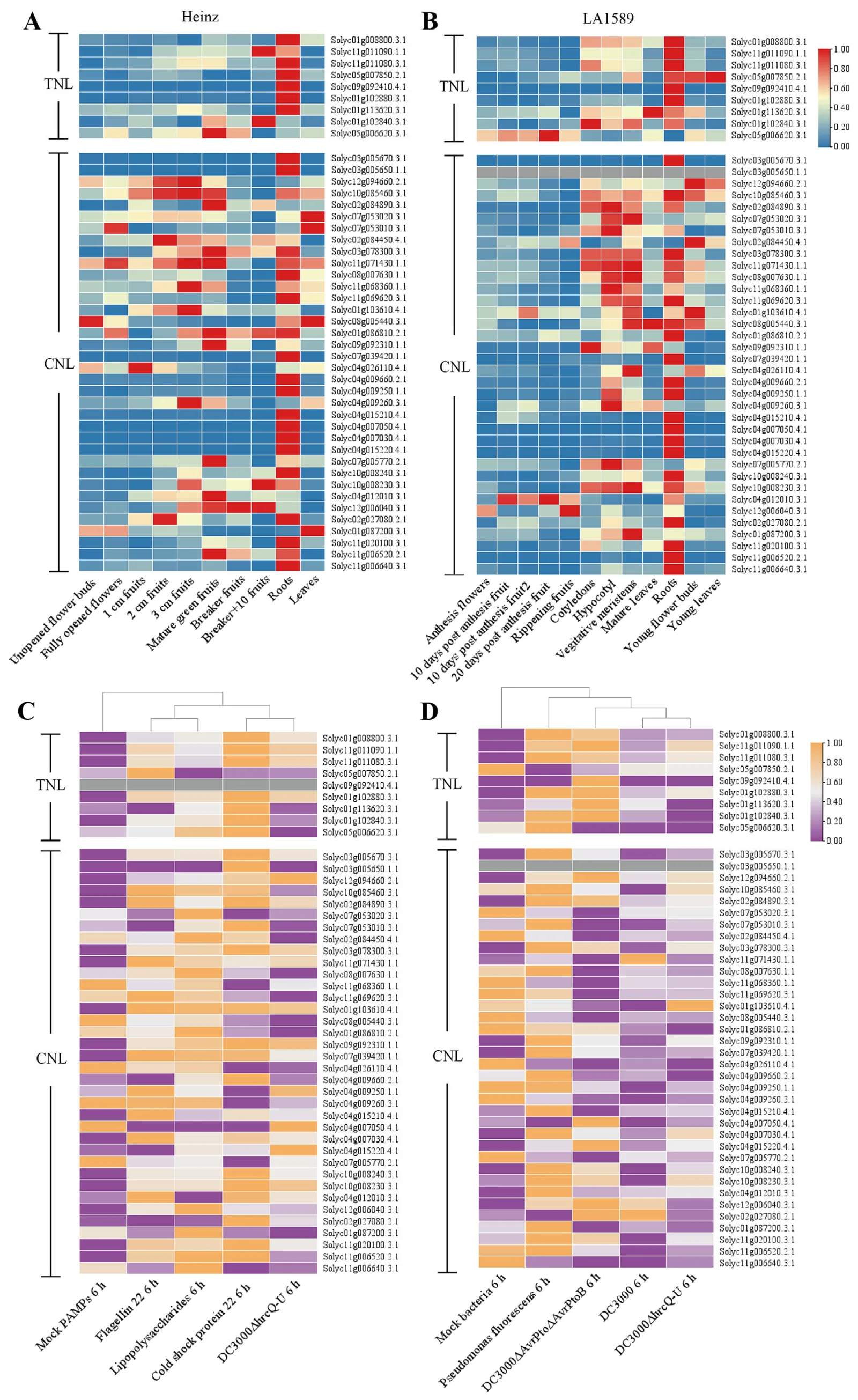
Heatmap of TNL and CNL gene expression levels in *S. lycopersicum* Heinz1706 and *S. pimpinellifolium* LA1589 (unit: RPKM). Genes with an average expression level greater than 1 across different tissues or developmental stages of Heinz were selected for analysis. (**A**) Expression levels of two types of NBS-LRR genes across various tissues of the cultivated Heinz variety are shown. (**B**) Expression patterns of the homologous genes in different tissues of the wild species *S. pimpinellifolium* LA1589 are presented. (**C**) Differential expression of TNL and CNL genes in response to various PAMPs, using mock PAMPs at 6 h as the reference group. FLG22: a conserved 22-amino acid peptide derived from bacterial flagellin, one of the most well-characterized PAMPs, known to robustly activate pattern-triggered immunity (PTI); Lipopolysaccharides: major structural components of the cell wall in Gram-negative bacteria; CSP22: a conserved peptide fragment from bacterial cold shock proteins, representing a typical PAMP; DC3000ΔhrcQ-U: a mutant strain lacking essential components of the type III secretion system (T3SS), which is unable to assemble a functional T3SS and thus cannot deliver effector proteins, triggering only PTI without suppressing it or inducing effector-triggered immunity (ETI), and is used to study pure PTI responses. (**D**) A heatmap showing the differential expression of TNL and CNL genes in response to various bacterial treatments, with mock bacteria at 6 h used as the reference group. DC3000: A model pathogen capable of infecting tomato and Arabidopsis, equipped with a complete T3SS that delivers effector proteins to suppress PTI and potentially activate ETI. DC3000ΔAvrPtoΔAvrPtoB: A mutant strain lacking two key effector proteins, AvrPto and AvrPtoB, resulting in a markedly diminished ability to suppress PTI, which typically leads to a stronger PTI response. Pseudomonas fluorescens: A fluorescent pseudomonad that may induce systemic resistance (ISR) in plants. In this context, it is used as a non-pathogenic control, as it generally lacks major virulence factors such as the T3SS, allowing for comparison with the pathogenic DC3000.

Transcriptome data for TNL and CNL genes in Heinz1706 were obtained from the Tomato Functional Genomics Database (Table S5). Both TNL and CNL genes displayed pronounced tissue-specific expression patterns, with consistently higher expression levels in roots across cultivated and wild accessions (Figure 4A,B). In Heinz1706, CNL genes showed broader expression across leaves, flowers, and fruits, whereas TNL expression was largely restricted to roots. In wild accession LA1589, expression levels in roots were generally higher, and both gene types exhibited expanded expression domains. Under immune treatments, CNL genes were strongly induced by multiple pathogen-associated molecular patterns (PAMPs), including Flagellin 22 (FLG22), Cold shock protein 22 (CSP22), and Lipopolysaccharides (LPS), whereas TNL genes responded more selectively, showing stronger induction under CSP22 treatment and effector-related bacterial treatments (Figure 4C,D). These results highlight distinct regulatory strategies of TNL and CNL genes in pattern-triggered and effector-triggered immune responses.

It should be noted that the gene expression profiling in the present study was merely performed based on publicly available transcriptome data through in silico analysis, without independent experimental verification such as qPCR under biotic and abiotic stress conditions. Further experimental validation is indispensable to solidify the functional interpretation and evolutionary characteristics of TNL and CNL genes. We therefore propose it as an important direction for future research.

## 4. Discussion

Taken together, our comparative analysis indicates that the TNL and CNL subfamilies of the tomato NBS-LRR gene family have followed distinct evolutionary and regulatory trajectories, rather than functioning as a structurally and functionally uniform gene pool. The consistent numerical dominance of CNL genes, together with their comparatively relaxed sequence constraint and broader expression, suggests a receptor class adapted for wide-ranging, rapidly evolving pathogen surveillance, whereas the stronger purifying selection and narrower expression of TNL genes point to a more specialized, functionally constrained role within the tomato immune repertoire. Because these interpretations are drawn from comparative genomic and transcriptomic evidence across a limited number of accessions rather than from direct functional validation, we present them as working hypotheses that provide a genomic framework for the future mechanistic and breeding-oriented studies discussed in the following subsections.

### 4.1. Integrated Comparative Framework for NBS-LRR Evolution in Tomato

In this study, we performed an integrated comparative analysis of the tomato NBS-LRR gene family by combining genome-wide identification, phylogenetic reconstruction, conserved domain and motif characterization, orthologous clustering, selection pressure analysis, and expression profiling across three representative tomato accessions (*S*. *chilense*, *S. lycopersicum* Heinz1706, and *S. lycopersicum* LA1464). By jointly analyzing one wild and two cultivated accessions, our study extends previous investigations that were largely based on a single reference genome [31] and provides a broader comparative context for understanding immune receptor evolution in tomato. By explicitly contrasting a wild accession with two cultivars representing different stages of domestication, our approach moves beyond the gene-count reporting offered by most previous single-genome surveys to directly ask whether evolutionary constraint and regulatory behavior differ systematically between the TNL and CNL subfamilies, and whether these differences track domestication history. Comparing wild and cultivated genomes in this way is important because domestication and breeding are known to alter both the genetic diversity and the selective pressures acting on disease-resistance gene families, with direct consequences for the resistance potential retained in modern cultivars. This comparative design allowed us to test whether the TNL/CNL divergence patterns detected in Heinz1706 also hold in an independently assembled wild genome and in a second, less intensively bred cultivar, thereby distinguishing genomic features that are conserved across the domestication gradient from those that differ between wild and cultivated backgrounds. This comparative design is particularly relevant for the NBS-LRR gene family, which is known for rapid evolution and lineage-specific expansion.

### 4.2. Subfamily Composition, Evolutionary Patterns, and Domestication-Related Differences

Genome-wide identification and phylogenetic analyses consistently revealed a strong predominance of nTNL genes, particularly CNLs, over TNL genes in tomato, with an approximate ratio of 5:1. This consistent ~5:1 ratio across three independently assembled genomes is compatible with, though does not directly demonstrate, a long-term evolutionary preference for CNL-type immune receptors in tomato: one plausible, though still speculative, explanation is that CNLs, as broad-spectrum immune receptors, may be more readily retained during tomato evolution to cope with a wide range of pathogen pressures, whereas TNLs, which appear to be under stricter functional constraints, may be restricted to a narrower set of specific immune responses; we present this as a hypothesis that requires functional validation rather than as an established mechanism. This distribution pattern contrasts with that observed in species such as Arabidopsis and soybean, which harbor expanded TNL repertoires [12], but closely resembles the gene composition reported in other Solanaceae species, including potato and tobacco [8,10]. The consistency of this subfamily bias across all three tomato accessions indicates that the predominance of nTNL genes is a stable genomic feature of tomato. Orthologous clustering analysis further revealed clear differences in gene similarity among the accessions. The two cultivated accessions, Heinz1706 and LA1464, shared a higher number of orthologous genes and exhibited greater overall sequence similarity. In contrast, the wild species *S. chilense* showed lower similarity to both cultivated accessions, although it was more closely related to the cherry tomato LA1464 than to the large-fruited Heinz1706, broadly consistent with recent population-level evidence that presence–absence variation in NLR genes in *S. chilense* is shaped largely by demographic history rather than by uniform domestication pressure [36]. These observations reflect differences in evolutionary history among the accessions and suggest that domestication and breeding may have influenced the composition and retention of specific NBS-LRR gene subsets in cultivated tomato [37].

The similarity of NBS-LRR gene composition between the two cultivated tomato lines (Heinz1706 and LA1464) was higher than that between the two and the wild tomato (*S. chilense*). This phenomenon is consistent with, but does not directly test, the hypothesis that domestication and subsequent breeding processes have exerted convergent selection pressure on the immune receptor gene pool, potentially resulting in the directional retention or loss of specific NBS-LRR gene variants associated with resistance to common pathogens in the cultivation environment, possibly at the expense of decreased genetic diversity relative to wild populations. We present this as a plausible interpretation of the observed pattern rather than as a demonstrated outcome of domestication, since the present comparative design (three accessions, with no explicit demographic modeling) cannot formally distinguish domestication-driven selection from other sources of divergence between wild and cultivated genomes.

### 4.3. Structural Divergence of TNL and CNL Proteins Revealed by Motif Composition

Conserved motif analysis revealed pronounced structural divergence between TNL and nTNL proteins. TNL proteins lacked several conserved motifs that were consistently present in nTNL proteins and also possessed subtype-specific motifs absent from nTNLs. The most prominent differences were located in the region corresponding to the second conserved motif within the NB-ARC domain, highlighting structural heterogeneity between the two subfamilies [38]. Importantly, this pattern of motif presence and absence was consistent across the three tomato accessions, despite differences in total gene number and evolutionary background. The recurrent loss or modification of specific motifs in TNL proteins, together with the conservation of motif architecture in CNL proteins, underscores clear structural differentiation between the two subfamilies [20]. Because several of the motifs that differ between TNL and CNL proteins map to regions implicated in nucleotide binding and downstream signaling (in the vicinity of the Walker A/B and MHD-like motifs of the NB-ARC domain), we speculate that these structural differences may underlie, at least in part, the distinct activation mechanisms and effector-recognition breadth of the two subfamilies discussed above, although this interpretation remains to be functionally tested.

### 4.4. Differential Evolutionary Constraints Acting on TNL and CNL Genes

Analysis of selective pressure revealed clear differences between TNL and CNL genes. Although TNL genes exhibited higher Ka and Ks values than CNL genes, their Ka/Ks ratios were significantly lower, indicating stronger purifying selection. This pattern reflects contrasting evolutionary dynamics between the two subfamilies at the sequence level. The coexistence of higher sequence divergence and lower Ka/Ks ratios in TNL genes suggests that a large proportion of sequence variation is selectively constrained. In contrast, CNL genes displayed relatively higher Ka/Ks values, reflecting a broader distribution of evolutionary rates within this subfamily. These quantitative differences in evolutionary parameters provide a detailed comparison of how selection pressures differ between TNL and CNL genes across tomato accessions. In addition, the inherent phylogenetic non-independence of NBS-LRR genes arising from chromosomal clustering and tandem duplication may bias conventional statistical tests; because more advanced phylogeny-aware and mixed-effects approaches could further refine these estimates (see Section 2.8), future studies may benefit from applying such methods to this gene family.

Disease resistance genes, particularly those of the NBS-LRR gene family, are known to be under positive selection [39,40]. In Arabidopsis, Mondragon-Palomino et al. have re-ported strong positive selection in many members of the NBS-LRR gene family [41]. We found that TNL genes are under stronger purifying selection, which may be because the TIR domain of the TNL receptor has a highly conserved function in signal transduction, and its mutation will lead to the failure of immune signaling [17]. In contrast, CNL genes have a higher evolutionary rate, possibly related to the diverse pathogens pressure faced by tomatoes during domestication; CNL, as a broad-spectrum immune receptor, requires faster evolution to adapt to new pathogens, a pattern broadly consistent with recent synteny-based evidence for widespread and recurrent TNL gene loss across flowering plants, which has been interpreted as reflecting stronger functional constraint on retained TNL loci [2].

### 4.5. Expression Divergence, Immune Regulation, and Future Perspectives

Expression profiling revealed pronounced divergence between TNL and CNL genes across tissues, developmental stages, and immune-related treatments. Both subfamilies exhibited consistently high expression in roots across cultivated and wild accessions, highlighting roots as a major site of NBS-LRR gene activity. Comparison of the expression heatmap with previously reported expression data indicates that, in cultivated tomato, CNL genes showed broader expression across leaves, flowers, and fruits, whereas TNL expression remained more spatially restricted [42]. In wild accessions, higher expression levels were observed in roots, and expression domains of both subfamilies were expanded relative to cultivated tomatoes.

Distinct expression responses were also observed under pathogen-associated molecular pattern (PAMP) and bacterial treatments. CNL genes responded broadly to multiple PAMPs, including FLG22, CSP22, and LPS, whereas TNL genes were preferentially induced by peptidic PAMPs and effector-deletion bacterial strains. These expression patterns provide a detailed description of regulatory differences between the two subfamilies under immune-related conditions.

Despite the comprehensive scope of this analysis, the study is limited by reliance on genome annotation quality and bioinformatics-based inference. Genes with incomplete or misannotated conserved domains may have been excluded, and motif-based predictions lack experimental validation. In particular, because LA1589 belongs to *S. pimpinellifolium* rather than *S. chilense*, differences in regulatory sequence, growth conditions, or sampling between these species may confound the comparison of expression patterns between wild and cultivated tomato; the wild-tomato expression patterns reported here should therefore be interpreted as representative of *S. pimpinellifolium* rather than as a direct proxy for *S. chilense*. In addition to these annotation- and surrogate-species-related caveats, the conclusions drawn here rest on descriptive comparative genomic and transcriptomic patterns across only three tomato accessions and have not been corroborated by direct experimental validation; the evolutionary and regulatory interpretations offered above should therefore be regarded as hypotheses to be tested rather than as established mechanisms.

Considered together, the structural, evolutionary, and regulatory results converge on a consistent picture: the reduced motif diversity, stronger purifying selection, and narrower expression domain of TNL genes point to a receptor subfamily maintained under tight functional constraint, while the more variable motif composition, relaxed selection, and broader expression of CNL genes are consistent with a subfamily favoring evolutionary flexibility and wide pathogen surveillance. Linking these independent lines of evidence in this way, rather than treating them as separate outputs of the analytical pipeline, strengthens the interpretation that TNL and CNL genes in tomato have diverged along coordinated structural, evolutionary, and regulatory axes, and provides a more mechanistically grounded rationale for prioritizing candidate genes in future resistance-breeding efforts.

Future studies integrating functional genomics approaches, pan-genomic analyses across additional Solanaceae species, and structural biology methods will further refine understanding of NBS-LRR gene diversification. Building directly on the present findings, future work should prioritize: (i) functional validation, such as gene silencing or CRISPR-based knockouts, of representative TNL and CNL candidates, to test whether their contrasting selective constraints correspond to differences in resistance breadth; (ii) expression profiling performed directly in *S. chilense*, rather than in the surrogate accession LA1589, to confirm the wild-tomato expression patterns reported here; and (iii) extension of the comparative framework to additional wild and cultivated Solanaceae genomes, to determine whether the TNL/CNL divergence patterns observed here generalize across the family. Together, the results presented here provide an extensive dataset and analytical framework for future investigations into tomato immune receptors and their application in disease-resistance breeding.

## 5. Conclusions

This study provides a comparative genomic and transcriptomic framework for understanding the evolutionary and regulatory divergence of the TNL and CNL subfamilies of NBS-LRR immune receptors in wild and cultivated tomato. Across three tomato accessions, CNL genes consistently outnumbered TNL genes by roughly 5:1 and displayed structurally distinct motif compositions, higher Ka/Ks ratios, broader tissue expression, and stronger PAMP-induced responses, whereas TNL genes were structurally more uniform, subject to stronger purifying selection, and more narrowly expressed, with induction concentrated under effector-related treatments. Together, these patterns suggest that the two receptor subfamilies may occupy complementary roles in tomato immune surveillance, with CNLs contributing broad, rapidly evolving pathogen detection and TNLs contributing a more constrained, specialized response; because these interpretations are based on computational and transcriptomic evidence rather than on functional experiments, they should be regarded as hypotheses for future testing. The principal contribution of this work is the joint, cross-accession comparison of TNL and CNL evolutionary constraint and expression behavior, which has not been systematically addressed in previous single-genome studies of the tomato NBS-LRR family. Its main limitations are the restriction to three accessions, the reliance on public genome annotations and transcriptomic datasets (including the use of *S. pimpinellifolium* LA1589 as a surrogate for *S. chilense* expression data), and the absence of experimental validation. Future work extending this comparison to additional wild and cultivated Solanaceae genomes, combined with functional validation of candidate TNL and CNL genes, will be needed to translate these genomic patterns into practical resources for disease-resistance breeding in tomato.

## Figures and Tables

**Table 1:** Classification and count of NBS-LRR genes in three tomato varieties.

Tomato Varieties	TNL	CNL	NL	Others	ALL	Proportion of TNL	Proportion of nTNL
*S. chilense*	33	89	47	7	176	18.75%	77.27%
*S. lycopersicum* Heinz1706	22	76	36	20	154	14.29%	72.73%
*S. lycopersicum* LA1464	25	87	41	21	173	14.45%	73.99%
All	80	252	124	48	503		

## Data Availability

Data derived from public domain resources: The data presented in this study are available in the NCBI RefSeq database at https://www.ncbi.nlm.nih.gov/refseq/ (accessed on 20 June 2025), reference numbers GCF_000499545.2 (*S. chilense*), GCF_000188115.4 (*S. lycopersicum* Heinz1706), and GCF_000390615.1 (*S. lycopersicum* LA1464). These data were derived from the following resources available in the public domain: the CAAS Tomato Pan-genome database (http://caastomato.biocloud.net/home [accessed on 18 June 2025]); the TomatoPangenome database (https://www.varnatech.cn/tomatoPan [accessed on 18 June 2025]); the NCBI RefSeq database (https://www.ncbi.nlm.nih.gov/refseq/ [accessed on 20 June 2025]); the Pfam database (https://www.ebi.ac.uk/interpro/entry/pfam/PF00931/ [accessed on 3 July 2025], HMM profile PF00931); the SMART domain annotation platform (http://smart.embl.de/ [accessed on 20 July 2025]); the Plant Reference Genes and Resistance Genes database PRGdb4.0 (https://prgdb.org/prgdb4/ [accessed on 22 July 2025], reference sequences PRGDB131, PRGDB140, and PRGDB141); the OrthoVenn3 platform (https://orthovenn3.bioinfotoolkits.net/ [accessed on 30 July 2025]); the MEME Suite (https://meme-suite.org/meme/ [accessed on 25 July 2025]); and the Tomato Functional Genomics Database (TFGD; http://ted.bti.cornell.edu [accessed on 16 August 2025]), Digital Expression Database, for RNA-seq expression data of *S. lycopersicum* Heinz1706 and *S. pimpinellifolium* LA1589. All other data supporting the findings of this study, including the complete NBS-LRR gene identification results, sequence alignments, and Ka/Ks values, are provided within the article and its Supplementary Materials (Tables S1–S5).

## Supplementary Materials

The following supporting information can be downloaded at: https://www.mdpi.com/article/10.3390/biology15161350/s1, Table S1: Complete NBS-LRR protein sequences of three *Solanum lycopersicum* accessions; Table S2: Conserved domain sequences of NBS-LRR proteins in three *Solanum lycopersicum* accessions; Table S3: Ten conserved motifs identified respectively in three *S. lycopersicum* accessions via the MEME website; Table S4: Ka, Ks, and Ka/Ks (ω) values of TNL and CNL orthologous gene clusters; Table S5: TNL and CNL gene expression levels of two *Solanum lycopersicum* accessions.
